# Pachydermoperiostosis Associated With a Rare SLCO2A1 Mutation: A Case Report and Literature Review

**DOI:** 10.7759/cureus.92731

**Published:** 2025-09-19

**Authors:** Maen Mohammad, Yousef Alnajjar, Enas Jondi, Mohammad Alsheikh, Adeeb Darras, Abdullah Hamamdah, Muaath Itmaizeh

**Affiliations:** 1 General Practice, Al-Quds University, Jerusalem, PSE; 2 Internal Medicine, Al-Makassed Islamic Charitable Society Hospital, Jerusalem, PSE; 3 Internal Medicine, Al-Quds University, Jerusalem, PSE

**Keywords:** case report, clubbing, pachydermoperiostosis, periostosis, primary hypertrophic osteoarthropathy

## Abstract

Pachydermoperiostosis (PDP), also known as primary hypertrophic osteoarthropathy, is a rare genetic disorder characterized by digital clubbing, periostosis, and pachydermia. It typically presents during adolescence or early adulthood and is commonly associated with mutations in the HPGD or SLCO2A1 genes. Due to its nonspecific clinical features, PDP is often misdiagnosed, resulting in delayed treatment. We report the case of a 26-year-old Arab male with an eight-year history of progressive facial skin thickening, hyperhidrosis, and digital clubbing. Laboratory investigations were unremarkable, while imaging revealed periostosis. Genetic analysis identified a SLCO2A1 mutation (c.563A>G, p.Gln188Arg).

Our patient experienced significant physical discomfort and psychological distress, with notable improvement following treatment with nonsteroidal anti-inflammatory drugs and corticosteroids. In our review of 246 PDP cases with SLCO2A1 mutations, the c.940+1G>A splice variant was most frequent. PDP shows marked male predominance (92.3%) and is most prevalent in Asian populations, particularly Chinese (53.3%). Most diagnoses occurred between ages ≤25 and 25-45 years. Common manifestations include digital clubbing (82.5%), pachydermia (74.4%), and hyperhidrosis (41.1%), with consanguinity reported in 29.7% of cases. Clinical overlap with rheumatologic and endocrine disorders often delays diagnosis. Genetic confirmation is essential for accurate identification. This case reinforces the importance of considering PDP in patients with unexplained digital clubbing and pachydermia. Early recognition and genetic confirmation of PDP can prevent unnecessary testing and guide effective treatment. Comprehensive, multidisciplinary care, including psychosocial support, is vital to optimize outcomes in this rare genetic disorder.

## Introduction

Pachydermoperiostosis (PDP) (Touraine-Solente-Gole syndrome) is a rare hereditary cause of hypertrophic osteoarthropathy. Hypertrophic osteoarthropathy can either be primary or secondary to underlying conditions affecting the pulmonary, cardiovascular, gastrointestinal, or other systems, with primary hypertrophic osteoarthropathy (PHO) accounting for less than 5% of all cases of hypertrophic osteoarthropathy [1,2]. PHO is classified based on genotype into three subtypes: PHOAR1 (15-hydroxyprostaglandin dehydrogenase (HPGD), autosomal recessive), PHOAR2 (solute carrier organic anion transporter family member 2A1 (SLCO2A1), autosomal recessive), and PHOAD (monoallelic SLCO2A1, autosomal dominant) [3]. It’s more common in adolescent males, with a male-female ratio of 7:1 [4].

PDP was first described in 1868 by Friedreich [5]. The precise pathophysiology of the disease remains poorly understood; however, current evidence shows that mutations in HPGD and SLCO2A1 lead to elevated prostaglandin E2 (PGE2) levels, through impaired degradation or transport, which underlie many features of PHO and provide the rationale for NSAID or COX-2 inhibitor therapy [6,7].

Herein, we report a rare case of a 26-year-old male patient whose presentation was highly suggestive of PDP and was diagnosed after a genetic study. A literature review providing data on SLCO2A1 mutations causing PDP is provided.

## Case presentation

A 26-year-old male Arab patient, previously in his usual state of health, presented to our clinic with complaints of progressive changes in his facial features and extremities over the past eight years. The initial symptoms began with thickening and folding of the forehead, which gradually extended to involve both cheeks and the entire face. Over time, the facial changes became more pronounced, with deeper skin folds and increased coarseness, as assessed during serial clinical examinations. Sweating was evaluated based on the patient’s subjective reports compared with their baseline sweating prior to symptom onset, which indicated a progressive increase.

Concurrently, the patient noticed a gradual enlargement of his fingers, initially affecting the proximal interphalangeal joints within the first one to two years, followed by involvement of the distal interphalangeal joints over the next two years, and later affecting the metacarpophalangeal joints and wrists during the subsequent three to four years. Additionally, the size of his toes, ankles, and knees gradually increased over the same eight-year period. However, no changes were noted in his elbows, shoulders, hips, or spine, and he denied experiencing any pain or erythema in the involved joints.

The patient did not report symptoms such as shortness of breath, orthopnea, palpitations, or tremors. He also reported no history of headaches, visual disturbances, or abnormal height increase. Additionally, a comprehensive review of cardiovascular, gastrointestinal, and neurological systems was also unremarkable. The patient’s past medical, surgical, and family histories were unremarkable. The patient was not taking any chronic medications. The patient finished high school. He is not married, does not smoke or drink alcohol, and does not take illicit drugs.

**Figure 1:**
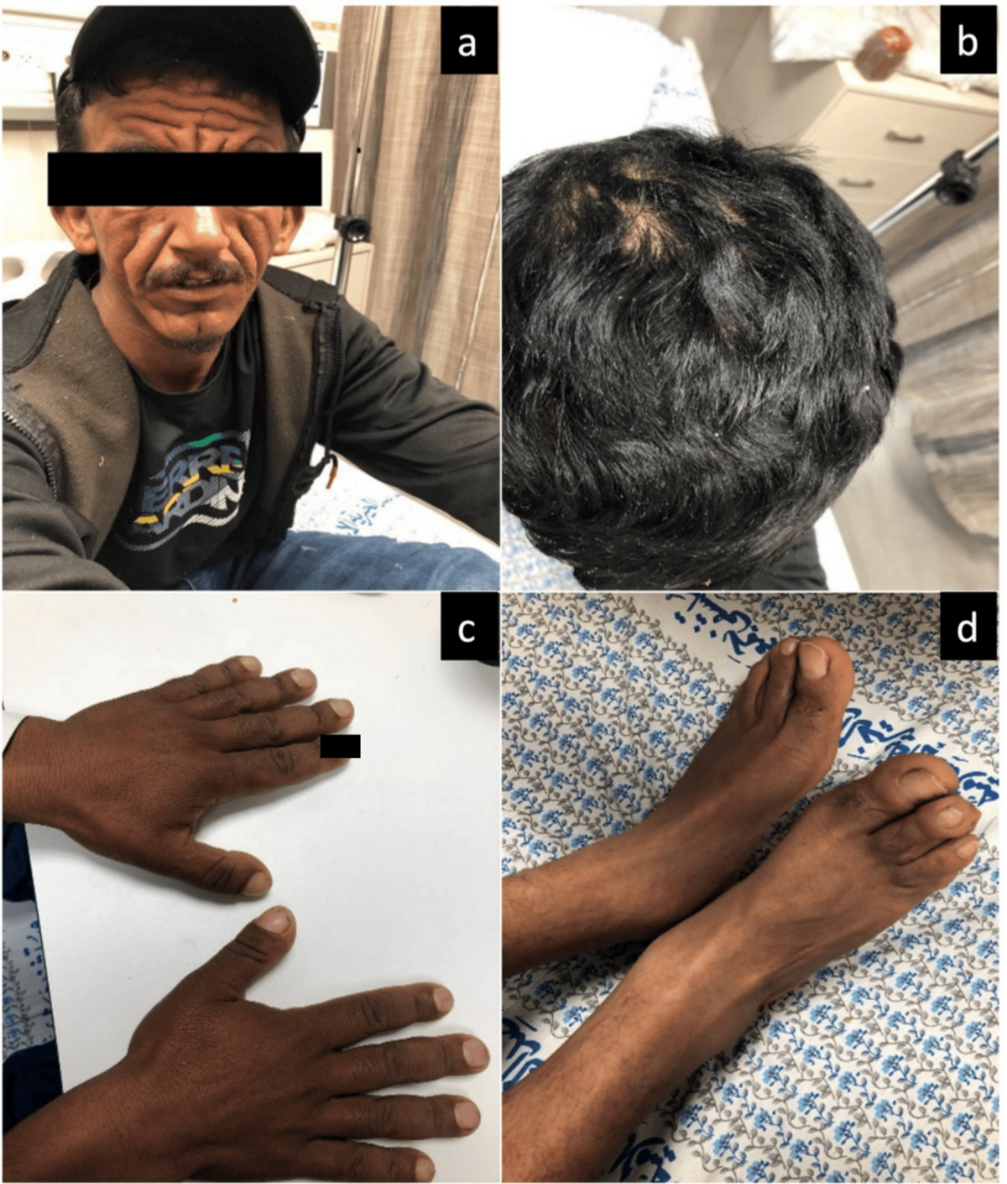
(a) Pronounced forehead skin folds with prominent nasolabial folds; (b) Presence of scalp dandruff; (c) Nail clubbing; (d) Swelling of both legs. Written informed consent has been provided by the patient to have the case details and any accompanying images published.

On physical examination, the patient appeared hemodynamically stable, alert, conscious, and oriented. Examination of the head and neck revealed marked skin folds in the forehead and cheeks, giving the patient a "leonine" appearance (Figure 1a). Notably, there was scalp dandruff, but no other dermatologic abnormalities were observed (Figure 1b). On musculoskeletal examination, there was grade 4 nail clubbing and swelling of the distal interphalangeal, proximal interphalangeal, metacarpophalangeal joints, and wrists (Figure 1c). Examination of the lower limbs revealed swelling of the bilateral knees, ankles, and toes (Figure 1d). Cardiovascular, respiratory, neurological, and thyroid physical examinations were otherwise unremarkable.

**Figure 2:**
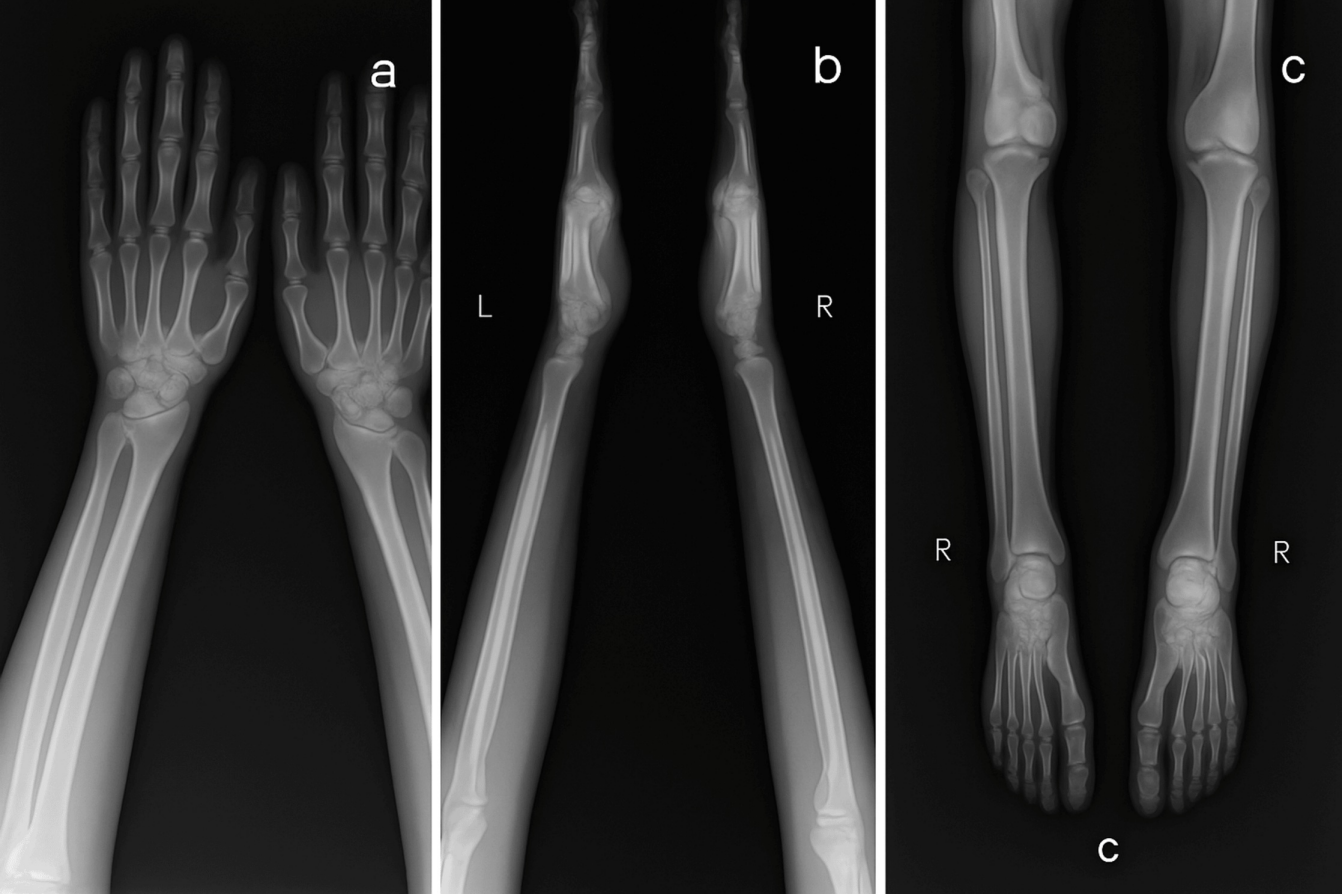
(a, b) Subperiosteal bone formation with enlargement of the phalanges, metacarpals, radius, and ulna. (c) Subperiosteal bone formation involving the bilateral distal tibiae and fibulae.

Radiographic imaging revealed bilateral subperiosteal bone formation in the phalanges, metacarpals, radius, and ulna (Figures 2a-2b), as well as increased cortical thickness due to subperiosteal bone formation in the tibias, fibulas, and tarsal bones (Figure 2c). To exclude secondary causes, the patient underwent a comprehensive work-up. Laboratory tests showed a white blood cell count of 9.6 x 10^9^/L, hemoglobin of 14.6 g/dL, and a platelet count of 378 x 10^9^/L. Liver, kidney, and thyroid function tests were normal. Investigations to rule out acromegaly, including an insulin-like growth factor-1 (IGF-1) level, were within normal limits. Rheumatoid factor (RF) and anti-cyclic citrullinated peptide (anti-CCP) antibodies were also negative. A computed tomography (CT) scan of the chest showed no evidence of pulmonary pathology. Echocardiography revealed no structural or functional cardiac abnormalities.

Over eight years, the patient exhibited progressive changes in facial and extremity features. After excluding secondary causes of hypertrophic osteoarthropathy through laboratory tests, imaging, and cardiovascular evaluation, clinical suspicion for a genetic form of PHO arose. Genomic amplification and direct sequencing of all exons of the HPGD and SLCO2A1 genes were performed on DNA extracted from the patient’s peripheral blood sample. The results revealed a homozygous c.563A>G (p.Gln188Arg) mutation in exon 4 of the SLCO2A1 gene, while no mutations were detected in the HPGD gene.

Over the past eight years, the patient’s social life has been significantly impacted by the progressive changes in his facial features and extremities. These physical changes led to considerable anxiety and a depressed mood, causing him to spend most of his time indoors. He consulted multiple doctors from different specialties before presenting to our hospital, and was distressed by not knowing his diagnosis. Although we advised the patient to seek psychiatric evaluation, he did not undergo a formal assessment. Upon receiving a diagnosis at our hospital, he experienced significant relief. The patient was treated with etoricoxib 90 mg orally once daily for six months to control pain and inflammation. Short-term corticosteroid therapy with prednisone 10 mg orally once daily for two weeks was administered during acute exacerbations. At his first follow-up visit one month after starting treatment, the patient reported a marked reduction in joint pain and facial discomfort. Examination revealed decreased joint tenderness and soft tissue swelling, with partial improvement in digital clubbing and pachydermia. Inflammatory markers (C-reactive protein (CRP) and erythrocyte sedimentation rate (ESR)) also showed a modest decline during follow-up. This case highlights the importance of expert medical care and the need for supportive, multidisciplinary management, including dermatology, rheumatology, and psychiatry, to address the emotional, psychological, and social challenges that patients with rare and complex conditions often face.

## Discussion

PDP, also known as PHO, is a rare genetic disorder with variable modes of inheritance described in the literature, the most commonly reported being the autosomal recessive mode in SLCO2A1 gene mutations [3,8]. Clinically, PDP is characterized by a combination of digital clubbing, pachyderma (skin thickening), excessive sweating, periostosis (inflammation of the periosteum), and enlargement of the hands and feet [9]. PDP can manifest in three clinical forms: the complete form, which includes both periostitis and pachydermia; the incomplete form, where bone changes are present without pachydermal features; and the forme fruste, which is characterized by pachydermal changes with little to no bone involvement.

PDP’s pathophysiology has not been completely understood. Literature suggests PDP results from elevated levels of prostaglandins, particularly PGE2. At the genetic level, mutations in either the HPGD gene, located on chromosome 4q34-q35, or the SLCO2A1 gene, which encodes a prostaglandin transporter, have been implicated in the pathogenesis of PDP [10-13]. The HPGD gene mutation leads to deficient activity of HPGD, the enzyme responsible for prostaglandin degradation, resulting in increased PGE2 levels. Similarly, mutations in the SLCO2A1 gene disrupt prostaglandin transport, contributing to elevated PGE2 concentrations and the subsequent clinical manifestations of the disease.

The definite pathophysiology explaining how increased levels of PGE2 lead to the clinical manifestations is not yet fully understood. In addition, many mutations have been reported in recent years, indicating that aspects of the disease are yet to be fully characterized. Our SLCO2A1 c.563A>G (p.Gln188Arg) variant, previously reported in a PDP cohort by Arcanjo et al., was identified in a homozygous proband with classic PDP [3]. This variant is absent from population databases. Our case provides additional clinical and imaging documentation that complements the existing cohort, illustrating the phenotypic spectrum associated with this rare, previously reported variant.

Being a rare condition, the exact number of reported cases of PDP remains undetermined, prompting us to conduct a thorough literature review. We searched PubMed for studies published since 2005, using the following keywords: "primary hypertrophic osteoarthropathy, pachydermoperiostosis, Touraine-Solente-Gole syndrome, and SLCO2A1." Our inclusion criteria were (1) studies published in English, (2) patients genetically diagnosed with a mutation in the SLCO2A1 gene causing PDP, and (3) studies that were fully accessible and provided relevant data. Exclusion criteria included (1) studies published in languages other than English, (2) studies published before 2005, (3) studies that were inaccessible or lacked necessary data, and (4) studies that identified SLCO2A1 mutations in the context of chronic enteropathy associated with SLCO2A1 (CEAS) only, without PDP. The collected data are presented in Table 1.

**Table 1:** Review of 246 reported cases of pachydermoperiostosis. References for the studies are cited within the main text of the manuscript. CEAS: chronic enteropathy associated with SLCO2A1; ESR: erythrocyte sedimentation rate; hsCRP: high-sensitivity C-reactive protein; H&E: hematoxylin and eosin; UC: ulcerative colitis; CT: computed tomography; MRI: magnetic resonance imaging; IP: interphalangeal; TMT: tarsometatarsal; MTP: metatarsophalangeal; NSAIDs: nonsteroidal anti-inflammatory drugs; ADHD: attention deficit hyperactivity disorder; PDA: patent ductus arteriosus

#	First author name	Year of publication	Sex (M/F)	Nationality/ethnicity	Symptoms first started? (age in years)	Age of diagnosis (years)	Excessive sweating	Progressive enlargement of hands and feet	Thickening of the skin of the face and scalp/pachyderma	Digital clubbing	Other manifestations/findings	Radiologic findings	Mutation type	Mutations site	Consanguinity (parents)	Previously suspected diagnosis?	Management
1	Pang Q [14]	2024	M	Chinese	-	-	-	-	-	-	-	-	Compound heterozygous	c.1375T>C c.850A>G	No	-	None
2			M	Chinese	-	-	-	-	-	-	-	-	Compound heterozygous	c.1375T>C c.850A>G	No	-	None
3			M	Chinese	-	-	-	-	-	-	-	-	Homozygous	c.1589G>A	No	-	None
4			M	Chinese	-	-	-	-	-	-	-	-	Compound heterozygous	c.940+1G>A c.1624C>T	No	-	None
5			M	Chinese	-	-	-	-	-	-	-	-	Heterozygous	c.830delT	No	-	None
6			M	Chinese	-	-	-	-	-	-	-	-	Compound heterozygous	c.823T>G c.1681C>T	No	-	None
7			M	Chinese	-	-	-	-	-	-	-	-	Homozygous	c.855delA	No	-	None
8			M	Chinese	-	-	-	-	-	-	-	-	Heterozygous	c.131_134GCCA>CCTGT	No	-	None
9			M	Chinese	-	-	-	-	-	-	-	-	Compound heterozygous	c.1136G>A c.1070A>G	No	-	None
10			M	Chinese	-	-	-	-	-	-	-	-	Compound heterozygous	c.941-1G>A c.1106G>A	No	-	None
11			M	Chinese	-	-	-	-	-	-	-	-	Compound heterozygous	c.611C>T c.1624C>T	No	-	None
12	Dai Y [15]	2024	M	Chinese	22	32	No	Yes	Yes	Yes	Recurrent lower abdominal pain, bloody and purulent stools Mild hypoalbuminemia and anemia ESR was elevated at 55 mm/h hsCRP was elevated at 23.41 mg/L Esophagogastroduodenoscopy revealed significant thickening of gastric folds in the cardia, fundus, body, antrum, and the descending duodenum Hematoxylin and eosin (H&E) stained gastric biopsies showed enlarged, densely packed glands Colonoscopy findings included diffuse mucosal edema, erosions, and purulent exudate in the sigmoid colon and rectum Colonic and rectal H&E stains showed inflammation and disrupted crypt architecture, consistent with ulcerative colitis (UC)	Periostosis, cortical thickening and diffuse increased bone density.	Homozygous	c.929A > G	No	-	mesalazine for enteropathy
13	Shang Q [16]	2024	M	Chinese	20	32	-	-	Yes	Yes	Ileus, diarrhea, hematochezia Reflux esophagitis Gastric polyps Ileal multiple strictures with annular ulcer Sigmoid colon isolated stricture with annular ulcer Congenital lymphatic dysplasia	Periostosis	Homozygous nonsense	c.1807C>T	No	-	-
14			M	Chinese	29	39	-	-	Yes	Yes	Ileus, diarrhea Gastric polyps Ileal multiple strictures with annular ulcer	Periostosis	Homozygous Frame shift	c.855delA	No	-	-
15			M	Chinese	25	27	-	-	Yes	Yes	Diarrhea, fever Gastric polyps Hypertrophic gastritis Diffuse mucosal erosions in colon and rectum	Periostosis	Homozygous Missense	c.929G>A	No	-	-
16			M	Chinese	18	26	-	-	Yes	Yes	Ileus, diarrhea Gastric polyps Ileal multiple strictures with annular ulcer	Periostosis	Compound heterozygous (Missense, Splicing site)	c.1106G>A c.941-1G>A	No	-	-
17			M	Chinese	5	31	-	-	Yes	Yes	Ileus, melena Gastric anastomotic ulcer Ileal multiple strictures with annular ulcer	Periostosis	Compound heterozygous (Frame shift, Missense)	c.1177delT c.1375T>C	No	-	-
18			M	Chinese	17	47	-	-	Yes	Yes	Ileus, pyloric obstruction Reflux esophagitis Gastric anastomotic ulcer Ileal multiple strictures with annular ulcer	Periostosis	Heterozygous Missense	c.1660G>A	No	-	-
19			M	Chinese	24	29	-	-	Yes	Yes	Ileus Hypertrophic gastritis Ileal multiple strictures with annular ulcer	Periostosis	Homozygous Nonsense	c.1771C>T	Yes	-	-
20	Niizeki H [17]	2024	43	Japanese	-	-	-	-	-	-	-	-	-	c.1279_1290del12	-	-	-
21	Long B [18]	2024	M	-	14	21	-	Yes	Yes	Yes	recurrent diarrhea enlargement of both knees and ankles greasy facial skin gastric pyloric gland adenoma foveolar‐type gastric adenoma	multiple periosteal reactions throughout the body and periarticular soft tissue swelling	homozygous	c.940 + 1G > A	-	-	-
22	Kimball TN [6]	2024	M	Mexico	-	-	-	-	-	-	CEAS	-		c.547G > A	-	-	-
23			M	Mexico	-	-	-	-	-	-	CEAS	-		c.1768del	-	-	-
24	Nakano Y [19]	2023	M	Japanese	14	20	Yes	Yes	Yes	Yes	recurrent knee hydrarthrosis	His knee showed thickening and fraying diaphysis in his lower extremities, without abnormality in the lumbar spine Cortical thickening of the long bones Symmetrical periosteal changes in the diaphysis of the fibula, tibia, metacarpals, metatarsals, and proximal phalanx in both hands and feet	Compound heterozygous (missense, splicing)	c.664G>A c.940+1G>A	No	Acromegaly	-
25	Zheng C [20]	2023	M	-	-	31	-	-	-	-	CEAS	-	Homozygous	c.290G>A	-	-	-
26	Bloch A [21]	2023	M	-	10	40	Yes	-	Yes	Yes	-	Periostosis	Homozygous	c.664G>A	-	-	-
27			M	-	22	27	No	-	Yes	Yes	-	Periostosis	Homozygous	c.861+2dupT	-	-	-
28			M	-	10	28	No	-	Yes	Yes	-	-	Homozygous	c.1241C>G	-	-	-
29			M	-	5	34	No	-	Yes	Yes	chronic diarrhea in childhood	-	Homozygous	c.1590C>A	-	-	-
30			M	-	15	28	Yes	-	Yes	Yes	-	Periostosis	Homozygous	c.1658delT	-	-	-
31			M	-	20	22	No	-	Yes	Yes	-	-	Homozygous	c.1682G>A	-	-	-
32			M	-	33	36	No	-	Yes	Yes	-	Periostosis	Heterozygous	c.234+1G>A	-	-	-
33			M	-	14	47	Yes	-	Yes	Yes	unilateral Deafness	Periostosis	Heterozygous	c.1523_1524del CT	-	-	-
34			M	-	40	41	No	-	No	Yes	-	Periostosis	Heterozygous	c.1625G>A	-	-	-
35			M	-	30	34	No	-	Yes	Yes	-	Periostosis	Heterozygous	c.31del	-	-	-
36	Chen B [22]	2023	M	Chinese	17	31	No	Yes	Yes	Yes	-	cortical hyperostosis in the distal tibia and fibula periostosis of the diaphysis in the distal left and right radius	Compound heterozygous (nonsense, splice-site)	c.96+4A>C in the exon-intron 2 boundary c.1807 C>T in exon 13 in the proband	No	-	-
37	Nicolau R [23]	2023	M	Portuguese	15	20	Yes	Yes	Yes	Yes	prolonged morning stiffness facial acne CRP: 35.5 mg/L ESR: 27 mm/h	soft tissue swelling, periosteal ossification and cortical thickening of the skull, phalanges, femur and toe acroosteolysis	Homozygous	c.1259G > T	No	-	naproxen 500 mg/day
38	Umair M [24]	2023	M	Pakistani	-	28	No	No	No	Yes	-	-	Homozygous Missense	c.155T>A	Yes	-	-
39			M	Pakistani	-	31	No	No	No	Yes	-	-	Homozygous Missense	c.155T>A	Yes	-	-
40	Pasumarthi D [25]	2023	M	South Indian	-	25	-	Yes	Yes	Yes	swelling and pain in knee joint heavy eyelids with ptosis, blepharoptosis Seborrhea	Periosteal reaction in long bones	Homozygous missense	c.614C>T	Yes	-	-
41			M	South Indian	-	23	Yes	Yes	Yes	Yes	recurrent intermittent fever pain and swelling of bilateral ankle joints	Periosteal reaction in long bones	Homozygous nonsense	c.529C>T	Yes	-	-
42			M	North Indian	-	20	-	Yes	Yes	Yes	bilateral swelling of ankle joints	Periosteal reaction in long bones	Homozygous Frameshift	c.1229_1238delTCCTTTGTGT	No	-	-
43	Dong Z [26]	2022	M	-	-	30	-	-	-	-	gastric cancer	-	-	-	-	-	-
44	Albawa'neh A [27]	2022	M	Emarati	-	22	-	Yes	Yes	Yes	symmetrical pain in his wrists, hand proximal interphalangeal (PIP) joints, ankles, and feet CRP: 15.44 mg/L	Periostosis MRI of the right ankle and foot showed an ankle effusion with a healing sprain of the posterior inferior tibiofibular ligament. There were effusions noted in the calcaneocuboid, naviculocuneiform, first tarsometatarsal (TMT), first metatarsophalangeal (MTP) joints, and the second–fifth distal interphalangeal (IP) joints and associated non-specific multifocal marrow edema.	Homozygous	c.289C>T	-	seronegative rheumatoid arthritis	etoricoxib 60 mg once daily
45	Biswas S [28]	2022	M	-	12	-	-	Yes	Yes	Yes	eyelids appeared to be swollen and drooping intermittent episodes of diarrhoea weight loss mild sensorineural hearing loss myelofibrosis	periosteitis upper gastrointestinal endoscopy: hypertrophic gastropathy	-	-	No	-	low dose steroids (prednisolone) etoricoxib retinoids
46			M	-	14	-	-	Yes	Yes	Yes	myelofibrosis	-	-	-	No	-	low dose steroids (prednisolone) etoricoxib retinoids vitamin A
47	Hong HS [29]	2022	F	Korean	21	39	-	-	No	Yes	CEAS Abdominal pain loose stool weight loss	periostosis	Homozygous splicing site Wild type	c.940+1G>A c.1807C>T, p.R603X	-	-	-
48			F	Korean	32	45	-	-	No	Yes	CEAS Abdominal pain loose stool weight loss	periostosis	Heterozygous Heterozygous	c.940+1G>A c.1807C>T, p.R603X	-	-	-
49			M	Korean	11	54	-	-	Yes	Yes	CEAS Abdominal pain loose stool GI bleeding	periostosis	Heterozygous Heterozygous	c.940+1G>A c.1807C>T, p.R603X	-	-	-
50			F	Korean	28	52	-	-	Yes	Yes	CEAS Abdominal pain loose stool	periostosis	Wild type Homozygous	c.940+1G>A c.1807C>T, p.R603X	-	-	-
51			M	Korean	15	29	-	-	Yes	Yes	CEAS Abdominal pain loose stool	periostosis	Heterozygous Heterozygous	c.940+1G>A c.1807C>T, p.R603X	-	-	-
52	Wang Q [30]	2022	M	-	16	18	Yes	No	Yes	Yes	Acne, seborrhea gynecomastia	Brain MRI scan showedscalp hypertrophy, which resembled petals. Radiography of both the legs showed periostitis	Compound heterozygous	-	-	-	200 mg of celecoxib
53	Ikeda K [31]	2022	M	-	15	35	-	-	Yes	Yes	limited range of motion in knee joints ptosis	periosteal thickening of extremities	-	-	-	-	-
54	Yousaf M [32]	2022	M	Pakistan	14	21	Yes	Yes	Yes	Yes	body aches and joint pains affecting large joints i.e., bilateral wrist and knee joints intermittent low-grade fever lethargy conjunctival pallor transfusion-dependent anemia acne hepatosplenomegaly myelofibrosis	-	Homozygous	c.664G>A	Yes	-	erythropoietin and cyclooxygenase-2 (COX 2) inhibitors
55	Jeyabaladevan S [33]	2022	M	Afghanistan	-	15	Yes	-	Yes	-	pain and swelling particularly affecting his knees and ankles, with large bilateral knee joint effusions ESR (70 mm/hr) CRP (22 mg l−1) allergic blepharo-conjuctivitis acne iron deficiency anaemia hyperplastic gastric polyps	periostosis was symmetrical and diffuse MRI of the left knee and ankle demonstrated circumferential periostosis, enlargement of the distal femur and distal tibia, synovial hypertrophy, and joint effusions	-	-	Yes	-	Knee aspirations intraarticular injections of 60 mg depomedrone
56	Kartal Baykan E [34]	2022	M	Turkey	13	16	Yes	No	Yes	No	pain in the elbow and knee over three years CRP: 11.4 mg/L	Bilateral knee and hand radiographs were considered normal	Homozygous frameshift	c.86delG (p.G29Afs*48)	-	Acromegaly	hydroxychloroquine was prescribed (200 mg twice daily, PO)
57			M	Turkey	12	17	Yes	Yes	Yes	Yes	swelling in knee joints, knee pain,	De novo bone formation and cortical thickening were detected on bilateral knee radiographs Irregular sub-periosteal de novo bone formation and cortical thickening were observed in the tibia, fibula, calcaneum, and talus on bilateral ankle radiographs	Homozygous nonsense	c.31C>T (p.Q11*)	Yes	-	selective Cox-2 inhibitor (oral meloxicam, 15 mg twice daily) steroid (oral methylprednisolone, 5 mg/day)
58	Marques P [35]	2022	M	-	-	26	Yes	Yes	Yes	Yes	prominent jaw and long eyelashes joint pain fatigue periodic episodes of watery diarrhea	-	Homozygous	-	-	Acromegaly	nonsteroid anti-inflammatory drugs zoledronate
59	Pang Q [36]	2022	M	Chinese	-	-	Yes	-	Yes	Yes	-	periostosis and acro-osteolysis	-	-	-	-	-
60			M	Chinese	-	-	Yes	-	Yes	Yes	-	periostosis and acro-osteolysis	-	-	-	-	-
61			M	Chinese	-	-	Yes	-	Yes	Yes	-	periostosis and acro-osteolysis	-	-	-	-	-
62			M	Chinese	-	-	Yes	-	Yes	Yes	-	periostosis and acro-osteolysis	-	-	-	-	-
63			M	Chinese	-	-	Yes	-	Yes	Yes	-	periostosis and acro-osteolysis	-	-	-	-	-
64			M	Chinese	-	-	Yes	-	Yes	Yes	-	periostosis and acro-osteolysis	-	-	-	-	-
65			M	Chinese	-	-	Yes	-	Yes	Yes	-	periostosis and acro-osteolysis	-	-	-	-	-
66			M	Chinese	-	-	Yes	-	Yes	Yes	-	periostosis and acro-osteolysis	-	-	-	-	-
67			M	Chinese	-	-	Yes	-	Yes	Yes	-	periostosis and acro-osteolysis	-	-	-	-	-
68			M	Chinese	-	-	Yes	-	Yes	Yes	-	periostosis and acro-osteolysis	-	-	-	-	-
69			M	Chinese	-	-	Yes	-	Yes	Yes	-	periostosis and acro-osteolysis	-	-	-	-	-
70			M	Chinese	-	-	Yes	-	Yes	Yes	-	periostosis and acro-osteolysis	-	-	-	-	-
71	Xu C [37]	2021	M	Chinese	15	16	Yes	Yes	Yes	Yes	seborrhea	soft tissue tumefaction and hyperostosis of metacarpal bones and phalanges irregular periosteal hypertrophy with subperiosteal new bone formation of bilateral tibia and fibula	Compound heterozygous	c.941-1G>A(splicing) c.754C>T(p.R252X)	No	-	Etoricoxib (60 mg per day)
72	Tran TH [38]	2021	M	Vietnamese	16	19	Yes	Yes	Yes	Yes	ESR: 24 mm/h CRP: 30.41 mg/L	Periostosis	Homozygous	c.669C>G	-	Rheumatoid arthritis	NSAIDs Hydroxychloroquine (both not effective)
73	Sun K [39]	2021	M	Chinese	-	33	-	Yes	Yes	Yes	Anemia intestinal perforation due to segmental coarctation of the intestinal tract splenomegaly portal hypertension splenic vein thickening intestinal ulcers decayed tooth	CT: partial intestinal aggregation and bowel wall thickening in the jejunum and ileum	Homozygous Heterozygous	c.210G>A (Homozygous) c.838C>T (Heterozygous)	No	-	-
74	Ishizuka T [12]	2021	M	-	12	41	-	Yes	Yes	Yes	ileus and gastric ulcer needing partial gastrectomy at 13 years old	hyperrtrophic periosteum at both femur, tibia, fibula	Homozygous splice-site	c.940+1G>A	-	rheumatoid arthritis	baricitinib (2mg/day),
75	Xu Y [40]	2021	-	Chinese	-	-	-	-	-	-	-	-	monoallelic	c.1660G>A	-	-	-
76			-	Chinese	-	-	-	-	-	-	-	-	monoallelic.	c.664G>A	-	-	-
77			-	Chinese	-	-	-	-	-	-	-	-	monoallelic	c.1065dupA	-	-	-
78			-	Chinese	-	-	-	-	-	-	-	-	monoallelic.	c.1293delT	-	-	-
79			-	Chinese	-	-	-	-	-	-	-	-	monoallelic.	c.1106G>A	-	-	-
80			-	Chinese	-	-	-	-	-	-	-	-	monoallelic	c.1807C>T	-	-	-
81			-	Chinese	-	-	-	-	-	-	-	-	monoallelic	c.1807C>T	-	-	-
82			-	Chinese	-	-	-	-	-	-	-	-	biallelic	c.664G>A c.621C>A	-	-	-
83			-	Chinese	-	-	-	-	-	-	-	-	biallelic	c.541G>C c.983T>C	-	-	-
84			-	Chinese	-	-	-	-	-	-	-	-	biallelic	c.1121C>T c.763G>A	-	-	-
85			-	Chinese	-	-	-	-	-	-	-	-	biallelic	c.1660G>A c.1814+1G>A	-	-	-
86			-	Chinese	-	-	-	-	-	-	-	-	biallelic	c.1807C>T c.1807C>T	-	-	-
87	Huang H [41]	2021	M	Chinese	12	27	-	-	Yes	Yes	Abdominal pain Anemia Hypoproteinemia intestinal ulcers CRP: 14.14 mg/L ESR: 25 mm/h	-	Homozygous	c.941-1G>A	No	-	-
88	Ishibashi M [42]	2021	M	-	-	18	-	-	Yes	Yes	-	Periostosis	Homozygous	c.940 þ 1G > A	-	-	-
89			M	-	-	25	-	-	Yes	Yes	-	Periostosis	Homozygous	c.940 þ 1G > A	-	-	-
90			M	-	-	29	-	-	Yes	Yes	-	Periostosis	Homozygous	c.940 þ 1G > A	-	-	-
91			M	-	-	32	-	-	Yes	Yes	-	Periostosis		c.940 þ 1G > A c.1807C > T	-	-	-
92			M	-	-	20	-	-	Yes	Yes	-	Periostosis	Homozygous	c.664G > A	-	-	-
93			M	-	-	50	-	-	Yes	Yes	-	Periostosis	Homozygous	c.664G > A	-	-	-
94			M	-	-	23	-	-	No	Yes	-	Periostosis		c.940 þ 1G > A c.1279_1290Δ12	-	-	-
95			M	-	-	29	-	-	No	Yes	-	Periostosis		c.940 þ 1G > A c.1279_1290Δ12	-	-	-
96			M	-	-	23	-	-	No	Yes	-	Periostosis	Homozygous	c.1279_1290Δ12	-	-	-
97	Oiwa T [43]	2021	M	-	16	20	No	Yes	Yes	Yes	Acne chronic diarrhea	Periostosis	Homozygous	c.940+1G>A, p.R288Gfs*7	-	-	Cosmetic surgery was performed on his forehead
98			M	-	12	25	No	Yes	Yes	Yes	duodenal ulcer Crohn’s disease	Periostosis	Homozygous	c.940+1G>A/p.R288Gfs*7	-	-	biweekly injection of adalimumab (80 mg)
99			M	-	15	21	No	Yes	Yes	Yes	facial acne ADHD	Periostosis	Homozygous	c.664G>A/p.G222R	-	-	atomoxetine for ADHD
100	Pang Q [44]	2020	M	Chinese	-	-	Yes	-	Yes	Yes	-	Periostosis	Homozygous	c.1589G>A	No	-	-
101			M	Chinese	-	-	Yes	-	Yes	Yes	-	Periostosis	Homozygous	c.855delA	No	-	-
102			M	Chinese	-	-	Yes	-	Yes	Yes	-	Periostosis	Heterozygous	c.131_134GCCA>CCTGT	No	-	-
103			M	Chinese	-	-	Yes	-	Yes	Yes	-	Periostosis	Compound heterozygous	c.941-1G>A c.1106G>A	No	-	-
104			M	Chinese	-	-	Yes	-	Yes	Yes	-	Periostosis	Compound heterozygous	c.1634delA c.823T>G	No	-	-
105			M	Chinese	-	-	Yes	-	Yes	Yes	-	Periostosis	Homozygous	c.855delA	No	-	-
106			M	Chinese	-	-	Yes	-	Yes	Yes	-	Periostosis	Compound heterozygous	c.823T>G c.1681C>T	No	-	-
107			M	Chinese	-	-	Yes	-	Yes	Yes	-	Periostosis	Compound heterozygous	c.823T>G c.129_130insC	No	-	-
108			M	Chinese	-	-	Yes	-	Yes	Yes	-	Periostosis	Compound heterozygous	c.1136G>A c.1070A>G	No	-	-
109			M	Chinese	-	-	Yes	-	Yes	Yes	-	Periostosis	Heterozygous	c.830delT	No	-	-
110	Tsuzuki Y [45]	2020	M	-	46	49	No	Yes	Yes	Yes	palpitation and shortness of breath upon exertion anemia hypoalbuminemia fecal occult blood test was positive intestinal capsule endoscopy demonstrated scattered round ulcers throughout the entire ileum CEAS with ileal ulcers	periostosis of the fingers and long bones in the limb Brain-CT revealed skin hypertrophy	Homozygous	c.1807C>T	-	-	RBC transfusion for severe anemia oral Fe tablets (100 mg/day) was commenced a few weeks later oral mesalazine (3,000 mg/day)
111	Sonoda A [46]	2020	M	-	-	65	-	-	Yes	Yes	anemia multiple circular and longitudinal ulcers throughout the entire small intestine gastric scarring CEAS	Periostosis	Compound heterozygous	c.940+1 G>A (splice site variant) c.1475 G>A	No	Gitelman syndrome Crohn's disease	-
112	Li N [47]	2020	M	Chinese	16	18	-	Yes	Yes	Yes	Anemia intermittent stomach pain	swelling of the soft tissue in both hands, knee joints, and feet symmetrical thickening of the first phalanx and first metatarsal	Homozygous	c.1807C>T	Yes	-	-
113	Torgutalp M [48]	2019	M	-	12	20	Yes	Yes	Yes	Yes	diffuse joint pain swelling and an hour of morning stiffness seborrheic eczema CRP: 57 mg/L ESR: 36 mm/h	neckchest-abdomen-pelvis computed tomography: normal Joint radiographs were similarly in accordance with inflammatory arthritis (especially erosions in wrists), but also revealed thickening in the mid-diaphyseal parts of the metacarpal and phalangeal bones, and cortical thickening on metaphysis of distal femur and proximal tibia Hypertrophic subperiosteal new bone formation and cortical irregularity with increased periosteal vascularity were determined by MRI of the femur	Homozygous	c.576C>G	-	Juvenile Idiopathic Arthritis:	acemetacin
114	Wang Q [49]	2019	M	Chinese	18	28	-	Yes	Yes	Yes	Diarrhea Hematochezia chronic superficial gastritis and fundic gland polyps scattered ulcers and hemorrhagic spots at the terminal ileum and colon	periostosis	Homozygous	c1807 C > T	-	-	etoricoxib 30 mg~ 60 mg once daily Partial enterectomy
115			M	Chinese	-	36	-	Yes	Yes	Yes	intermittent abdominal colic, diarrhea, and anemia	periostosis	Homozygous	c.855delA	-	-	etoricoxib 60 mg once daily mesalazine (3 g/day) for 3 months prednisone (0.8 g/kg/day) for 1 month Partial enterectomy
116	Xiao J [50]	2019	M	Chinese	14	-	-	Yes	Yes	Yes	-	-	Compound heterozygous	c.310G>A c.861+1G>A	-	-	-
117			M	Chinese	-	-	-	Yes	No	Yes	-	-	Heterozygous	c.310G>A	-	-	-
118	Li X [51]	2019	M	Tibetan	15	19	-	Yes	Yes	Yes	hematemesis seborrhea and acne	X‑ray of the extremities demonstrated enlarged diaphysis, soft tissue swelling and periosteal proliferation Gastroscopy revealed hypertrophic gastric folds with multiple ulcers in gastric antrum	Homozygous nonsense	c.1807C>T	No	-	-
119	Jiang Y [52]	2019	M	Chinese	16	33	Yes	Yes	Yes	Yes	pain and swelling of knees weakness of the extremities diarrhea serum potassium of 3.3 mmol/L	periosteal hyperostosis of tibia and fibula periosteal hyperostosis of phalanges	Compound heterozygous	c.850 A > G c.1375 T > C	No	-	etoricoxib 60 mg qd oral potassium supplements
120			M	Chinese	20	35	-	Yes	Yes	Yes	serum potassium was 3.4 mmol/L	periosteal hyperostosis of tibia and fibula periosteal hyperostosis of phalanges	Compound heterozygous	c.850 A > G c.1375 T > C	No	-	-
121	Yuan L [53]	2018	M	Chinese	-	43	Yes	Yes	Yes	Yes	Arthralgia of large joints	Periostosis	Heterozygous frameshift Heterozygous missense	c.122delC c.1781G>A	No	-	-
122			M	Chinese	-	37	Yes	Yes	Yes	Yes	-	Periostosis	Heterozygous frameshift Heterozygous missense	c.122delC c.1781G>A	No	-	-
123			M	Chinese	-	25	Yes	Yes	Yes	Yes	Arthralgia of large joints	Periostosis	Homozygous missense	c.1681C>T c.1681C>T	No	-	-
124			M	Chinese	-	33	Yes	Yes	Yes	Yes	-	Periostosis	Heterozygous missense Heterozygous	c.440G>A c.940+1G>A	No	-	-
125			M	Chinese	-	38	Yes	Yes	Yes	Yes	-	Periostosis	Compound heterozygous	c.724+1G>A c.940+1G>A	No	-	-
126	Umeno J [54]	2018	M	Japanese	-	26	-	-	Yes	Yes	CEAS Two lesions of ulceration with stenosis were observed in the ileum	Periostosis	Compound heterozygous	c.547G > A c.940 + 1G > A	-	-	-
127			M	Japanese	-	-	-	-	Yes	Yes	CEAS	Periostosis	-	-	-	-	-
128			M	Japanese	-	-	-	-	Yes	Yes	CEAS	Periostosis	-	-	-	-	-
129			M	Japanese	-	-	-	-	Yes	Yes	CEAS	Periostosis	-	-	-	-	-
130			M	Japanese	-	-	-	-	Yes	Yes	CEAS	Periostosis	-	-	-	-	-
131	Alessandrella A [55]	2018	M	Morrocan	13	17	Yes	Yes	Yes	Yes	effusion in his knees kyphoscoliosis	metaphysis flaring of the distal femur and proximal tibia swelling of the periarticular soft tissue and cortical thicken- ing of the first metatarsal bilaterally	Homozygous	c.1658delT	No	-	hydroxychloroquine (400 mg per day).
132	Sun F [56]	2018	M	Chinese	17	23	-	Yes	Yes	Yes	intermittent watery diarrhea	Periostosis	Homogenous missense	c.547G>A	Yes	-	Etoricoxib (60 mg/d)
133	Villarreal-Martínez A [57]	2018	M	Mexican	13	23	-	-	Yes	Yes	severe acne and hyperseborrhea	periosteal bone formation circumferentially along the shaft of the tibia and fibula as well as cortical thickening at the distal ends.	Compound heterozygous	c.572G>C c.1186G>A	-	-	-
134			M	Mexican	13	24	-	-	Yes	Yes	bilateral knee pain, swelling and reduced range of movement Ota nevus on the left side of the face prognathism	subperiosteal reaction in the lateral condyle of the femur and tibia	Homozygous	c.96+5>A	-	-	-
135	Li SS [58]	2017	M	Chinese	-	31.6	Yes	-	Yes	Yes	Seborrhae Arthralgia Watery diarrhea	Periostosis	Homozygous	c.941-1G>A	-	-	etoricoxib (Arcoxia, 60 mg/day; Merck & Co) for 6 months
136			M	Chinese	-	31.7	Yes	-	Yes	Yes	Seborrhae Arthralgia Watery diarrhea	Periostosis	-	c.941-2A>G c.1406C>T	-	-	etoricoxib (Arcoxia, 60 mg/day; Merck & Co) for 6 months
137			M	Chinese	-	25.6	Yes	-	Yes	Yes	Seborrhae Watery diarrhea	Periostosis	Homozygous	c.1406C>T	-	-	etoricoxib (Arcoxia, 60 mg/day; Merck & Co) for 6 months
138			M	Chinese	-	29.5	Yes	-	Yes	Yes	Seborrhae	Periostosis	-	c.1069T>C c.1406C>T	-	-	etoricoxib (Arcoxia, 60 mg/day; Merck & Co) for 6 months
139			M	Chinese	-	35.9	No	-	Yes	Yes	-	Periostosis	-	c.440G>A c.1624C>T	-	-	etoricoxib (Arcoxia, 60 mg/day; Merck & Co) for 6 months
140			M	Chinese	-	24.8	Yes	-	Yes	Yes	Seborrhae Watery diarrhea	Periostosis	Homozygous	c.1602C>A	-	-	etoricoxib (Arcoxia, 60 mg/day; Merck & Co) for 6 months
141			M	Chinese	-	21.9	Yes	-	No	Yes	Seborrhae Arthralgia Watery diarrhea	Periostosis	Homozygous	c.1771C>T	-	-	etoricoxib (Arcoxia, 60 mg/day; Merck & Co) for 6 months
142			M	Chinese	-	19.3	Yes	-	Yes	Yes	Seborrhae Arthralgia Watery diarrhea	Periostosis	-	c.178G>A c.547G>A	-	-	etoricoxib (Arcoxia, 60 mg/day; Merck & Co) for 6 months
143			M	Chinese	-	29.4	Yes	-	Yes	Yes	Seborrhae	Periostosis	Homozygous	deletion(NC_000003 133692071-133693956)	-	-	etoricoxib (Arcoxia, 60 mg/day; Merck & Co) for 6 months
144			M	Chinese	-	18.4	Yes	-	Yes	No	Seborrhae	Periostosis	-	c.178G>A c.1660G>A	-	-	etoricoxib (Arcoxia, 60 mg/day; Merck & Co) for 6 months
145			M	Chinese	-	24	Yes	-	Yes	Yes	Seborrhae	Periostosis	-	c.759G>A c.764G>A	-	-	etoricoxib (Arcoxia, 60 mg/day; Merck & Co) for 6 months
146			M	Chinese	-	19.6	Yes	-	Yes	Yes	Arthralgia	Periostosis	-	c.1378G>A c.611C>T	-	-	etoricoxib (Arcoxia, 60 mg/day; Merck & Co) for 6 months
147			M	Chinese	-	33.4	Yes	-	Yes	Yes	Seborrhae	Periostosis	-	c.1406C>T c.1771C>T	-	-	etoricoxib (Arcoxia, 60 mg/day; Merck & Co) for 6 months
148			M	Chinese	-	24.9	Yes	-	No	Yes	Seborrhae Watery diarrhea	Periostosis	-	c.1292dupC c.1456C>A	-	-	etoricoxib (Arcoxia, 60 mg/day; Merck & Co) for 6 months
149			M	Chinese	-	21.7	No	-	Yes	Yes	Seborrhae	Periostosis	Homozygous	c.1807C>T	-	-	etoricoxib (Arcoxia, 60 mg/day; Merck & Co) for 6 months
150			M	Chinese	-	27.5	Yes	-	Yes	Yes	Seborrhae Arthralgia	Periostosis	Homozygous	c.941-1G>A	-	-	etoricoxib (Arcoxia, 60 mg/day; Merck & Co) for 6 months
151			M	Chinese	-	21.9	No	-	Yes	Yes	-	Periostosis	-	c.1624C>T c.1807C>T	-	-	etoricoxib (Arcoxia, 60 mg/day; Merck & Co) for 6 months
152			M	Chinese	-	26.8	Yes	-	Yes	Yes	Seborrhae Watery diarrhea	Periostosis	Homozygous	c.440G>A	-	-	etoricoxib (Arcoxia, 60 mg/day; Merck & Co) for 6 months
153			M	Chinese	-	21.2	Yes	-	Yes	Yes	Seborrhae	Periostosis	-	c.754C>T c.1106G>A	-	-	etoricoxib (Arcoxia, 60 mg/day; Merck & Co) for 6 months
154			M	Chinese	-	21.1	No	-	Yes	Yes	Seborrhae Arthralgia Watery diarrhea	Periostosis	-	c.1287C>A c.1406C>T	-	-	etoricoxib (Arcoxia, 60 mg/day; Merck & Co) for 6 months
155			M	Chinese	-	29.5	Yes	-	Yes	Yes	Seborrhae Watery diarrhea	Periostosis	Homozygous	c.838C>T	-	-	etoricoxib (Arcoxia, 60 mg/day; Merck & Co) for 6 months
156			M	Chinese	-	25	Yes	-	Yes	Yes	Seborrhae Arthralgia Watery diarrhea	Periostosis	Homozygous	c.838C>T	-	-	etoricoxib (Arcoxia, 60 mg/day; Merck & Co) for 6 months
157			M	Chinese	-	21	Yes	-	Yes	Yes	Watery diarrhea	Periostosis	Homozygous	c.838C>T	-	-	etoricoxib (Arcoxia, 60 mg/day; Merck & Co) for 6 months
158			M	Chinese	-	24.2	No	-	Yes	Yes	Seborrhea, watery diarrhea	Periostosis	-	c.178G>A c.310G>A	-	-	etoricoxib (Arcoxia, 60 mg/day; Merck & Co) for 6 months
159			M	Chinese	-	24.9	No	-	Yes	Yes	Seborrhea, arthralgia, watery diarrhea	Periostosis	Heterozygous	c.1293delT	-	-	etoricoxib (Arcoxia, 60 mg/day; Merck & Co) for 6 months
160			M	Chinese	-	25.2	Yes	-	Yes	Yes	Seborrhea	Periostosis	Homozygous	c.1372G>T	-	-	etoricoxib (Arcoxia, 60 mg/day; Merck & Co) for 6 months
161			M	Chinese	-	19.9	Yes	-	Yes	Yes	Seborrhea	Periostosis	-	c.289C>T c.1106G>A	-	-	etoricoxib (Arcoxia, 60 mg/day; Merck & Co) for 6 months
162			M	Chinese	-	42.4	Yes	-	Yes	Yes	Seborrhea, watery diarrhea	Periostosis	Heterozygous	c.1660G>A	-	-	etoricoxib (Arcoxia, 60 mg/day; Merck & Co) for 6 months
163			M	Chinese	-	24.9	No	-	Yes	Yes	Seborrhea, arthralgia	Periostosis	-	c.96+4A>C c.565C>T	-	-	etoricoxib (Arcoxia, 60 mg/day; Merck & Co) for 6 months
164			M	Chinese	-	35.9	Yes	-	Yes	Yes	Seborrhea, watery diarrhea	Periostosis	Homozygous	c.941-1G>A	-	-	etoricoxib (Arcoxia, 60 mg/day; Merck & Co) for 6 months
165			M	Chinese	-	30.1	Yes	-	No	Yes	Seborrhea, watery diarrhea	Periostosis	Heterozygous	c.664G>A	-	-	etoricoxib (Arcoxia, 60 mg/day; Merck & Co) for 6 months
166			M	Chinese	-	22.2	Yes	-	Yes	Yes	Seborrhea	Periostosis	-	c.656C>T c.1839C>A	-	-	etoricoxib (Arcoxia, 60 mg/day; Merck & Co) for 6 months
167			M	Chinese	-	25	Yes	-	Yes	Yes	Seborrhea	Periostosis	-	c.1106-1G>A c.1807C>T	-	-	etoricoxib (Arcoxia, 60 mg/day; Merck & Co) for 6 months
168			M	Chinese	-	24	Yes	-	Yes	Yes	Seborrhea	Periostosis	-	c.861+2T>C c.1095C>A	-	-	etoricoxib (Arcoxia, 60 mg/day; Merck & Co) for 6 months
169			M	Chinese	-	51	No	-	Yes	Yes	Seborrhea, arthralgia, watery diarrhea	Periostosis	-	c.621C>A c.664G>A	-	-	etoricoxib (Arcoxia, 60 mg/day; Merck & Co) for 6 months
170			M	Chinese	-	22.7	Yes	-	Yes	Yes	Seborrhea, watery diarrhea	Periostosis	-	c.440G>A c.1370C>T	-	-	etoricoxib (Arcoxia, 60 mg/day; Merck & Co) for 6 months
171	Ma W [59]	2017	M	-	-	25	Yes	Yes	Yes	Yes	Cystic acne folliculitis	Periostosis	Homozygous missense	c.101T > C	-	Acromegaly	-
172	Guo T [60]	2017	M	Chinese	18	20	-	-	Yes	Yes	-	Periostosis	Compound heterozygous	c.349 delC (frameshift deletion) c.1286A>G (missence mutation)	No	-	-
173	Karimova MM [61]	2017	M	Uzbek	-	24	-	Yes	Yes	Yes	Excessive perspiration, generalized fatigue, scattered cafe-au-lait patches on abdomen	-	Homozygous	c.764G.A	No	Acromegaly	-
174	Mangupli R [8]	2017	M	Venezuelan	12	20	-	Yes	Yes	Yes	Knee pain (extensive swelling and effusions bilaterally)	subperiostial bone formation with bone density and cortical thickening	Homozygous	c.830delT	-	Acromegaly	-
175	Tanese K [62]	2017	M	Japanese	14	21	-	-	Yes	Yes	-	Periostosis		c.940+1G>A c.1279_1290del12	-	-	-
176			M	Japanese	13	21	-	-	Yes	Yes	-	Periostosis		c.940+1G>A c.1279_1290del12	-	-	-
177			M	Japanese	13	24	-	-	-	-	-	-		c.940+1G>A c.1279_1290del12	-	-	-
178	Shah K [63]	2017	M	Pakistani			No	No	No	Yes	-	-	Homozygous	c.1A>G	-	-	-
179			M	Pakistani			No	No	No	Yes	-	-	Homozygous	c.1A>G	-	-	-
180			F	Pakistani			No	No	No	Yes	-	-	Homozygous	c.1A>G	-	-	-
181	Huang H [64]	2017	M	Chinese	19	32	Yes	Yes	Yes	Yes	Gastric mucosa hyperplasia	Periostosis	-	c.1106G>A	Yes	-	-
182			M	Chinese	18	27	Yes	Yes	Yes	Yes	Gastric mucosa hyperplasia	Periostosis	-	c.1106G>A c.1106G>A	Yes	-	-
183			M	Chinese	21	33	Yes	Yes	Yes	Yes	Gastric mucosa hyperplasia	Periostosis	-	c.1106G>A c.941-1G>A	No	-	-
184			M	Chinese	15	24	Yes	Yes	Yes	Yes	Gastric mucosa hyperplasia	Periostosis	-	c.1771C>T c.1406C>T	No	-	-
185			M	Chinese	19	33	Yes	Yes	Yes	Yes	Gastric mucosa hyperplasia	Periostosis	-	c.1602C>A	No	-	-
186			M	Chinese	14	33	Yes	Yes	Yes	Yes	Gastric mucosa hyperplasia	Periostosis	-	c.611C>T c.96+4A>C	No	-	-
187			M	Chinese	16	32	Yes	Yes	Yes	Yes	Gastric mucosa hyperplasia	Periostosis	-	c.1069T>C	No	-	-
188	Lee S [13]	2016	M	Korean	19	56	Yes	-	Yes	Yes	Seborrhoea and eczema, acne	Periostosis	-	c.302T>G			
189			M	Korean	17	54	No	-	Yes	Yes	Seborrhoea and eczema, acne, hydrarthrosis	Periostosis	-	c.302T>G			
190			M	Korean	20	52	No	-	Yes	Yes	Seborrhoea and eczema, acne, hydrarthrosis	Periostosis	-	c.302T>G			
191			M	Korean	17	19	Yes	-	Yes	Yes	Seborrhoea and eczema, acne, hydrarthrosis PDA	Periostosis	-	c.940+1G>A c.1807C>T			
192			M	Korean	-	23	Yes	-	Yes	Yes	Seborrhoea and eczema, acne, hydrarthrosis	Periostosis	-	c.940+1G>A c.940+1G>A			
193			M	Korean	13	19	Yes	-	Yes	Yes	Seborrhoea and eczema, acne, hydrarthrosis PDA	Periostosis	-	c.940+1G>A c.940+1G>A			
194	Saadeh D [65]	2015	M	Lebanese	-	22	-	Yes	Yes	Yes	Arthralgia	Periostosis	-	c.838C>T c.838C>T	-	-	-
195			M	Lebanese	-	24	-	Yes	Yes	Yes	Arthralgia	-	-	c.838C>T c.838C>T	-	-	-
196	Ayoub N [66]	2015	M	Saudi	-	23	Yes	Yes	Yes	Yes	Arthralgia, seborrhoea, facial acne	Periostosis	Homozygous	c.1016C>T	-	-	-
197	Giancane G [67]	2015	M	-	13	21	Yes	Yes	Yes	Yes	Seborrhoea, aplastic marrow	-	Compound heterozygous	c.754C>T c.794C>G	-	JIA	Ibuprofen 400 mg 3 times per day low dose steroid therapy oxybutynin 2.5 mg 3 times per day
198	Minakawa S [68]	2015	M	Japanese	12	15	Yes	-	Yes	Yes	Seborrhea, arthralgia	Periostosis	Compound heterozygous	c.940+1G>A c.1279_1290del12	-	-	-
199	Kim HJ [69]	2015	M	Korean	13	19	-	-	Yes	Yes	Severe acne, arthralgia, watery diarrhea	Knee joint radiology revealed periosteal reaction with suspicious diaphyseal widening along the femur and tibia	Homozygous	c.940 + 1G > A	No	-	-
200	Madruga Dias JA [70]	2014	M	African	16	26	-	Yes	Yes	Yes	Arthralgia	Periostosis	Homozygous	c.940+1G>A c.940+1G>A	No	-	etoricoxib 60 mg/day
201	Niizeki H [71]	2014	F	Japanese	43	67	No	No	No	Yes	Myelopathy, arthralgia	Periostosis	Compound heterozygous	c.1279G>A c.1807C>T	-	-	-
202	Niizeki H [72]	2014	M	Japanese	15	19	Yes	Yes	Yes	Yes	Acne, seborrhea, and eczema	Periostosis	Compound heterozygous	c.940+1G>A c.1279_1290del12	No	-	-
203			M	Japanese	16	21	Yes	Yes	Yes	Yes	Acne, seborrhea, and eczema	Periostosis	Compound heterozygous	c.1807C>T c.754C > T	No	-	-
204			M	Japanese	14	20	Yes	Yes	Yes	Yes	Acne	Periostosis	Compound heterozygous	c.940+1G>A c.421G > T	No	-	-
205			M	Japanese	14	20	No	Yes	Yes	Yes	Acne, seborrhea, and eczema	Periostosis	Compound heterozygous	c.940+1G>A c.1807C > T	No	-	-
206	Cheng R [73]	2013	M	Chinese	20	25	Yes	Yes	Yes	Yes	Arthralgia, seborrhea, gastric polyps, and erosive gastritis	Periostosis	Compound heterozygous	c.547G>A c.1807C>T	No	-	-
207			M	Chinese	18	37	-	-	Yes	Yes	Arthralgia Seborrhae	Periostosis	Compound heterozygous	c.940+1G>A c.1602C>A	No	-	-
208	Zhang Z [74]	2013	M	Chinese	19	22	Yes	Yes	Yes	Yes	Recurrent blepharitis	periosteal overgrowth of the long bones	Compound heterozygous	c.940 + 1G > A c.1602C > A (missense)	No	-	-
209	Zhang Z [75]	2013	M	Chinese	16	36	No	Yes	Yes	Yes	Stomachache, watery diarrhea, severe anemia, and hypoalbuminemia	periosteal overgrowth of the long bones	Homozygous	c.855delA	No	-	-
210			F	Chinese	-	47	No	-	-	-	Watery diarrhea, anemia, and hypoalbuminemia	-	Homozygous	c.855delA	No	-	-
211			F	Chinese	-	42	No	-	-	-	Watery diarrhea, anemia, and hypoalbuminemia	-	Homozygous	c.855delA	No	-	-
212			M	Chinese	-	23	Yes	Yes	Yes	Yes	Seborrhea, acne, watery diarrhea	periosteal overgrowth of the long bones	Homozygous	c.1106G>A	Yes	-	-
213			M	Chinese	10	26	Yes	Yes	Yes	Yes	Stomachache, watery diarrhea, recurrent blepharitis	periosteal overgrowth of the long bones	Homozygous	c.1393G>A	Yes	-	-
214			M	Chinese	16	18	No	Yes	Yes	Yes	Seborrhea, acne, watery diarrhea	periosteal overgrowth of the long bones	Compound heterozygous	c.493G>T c.1136G>A	No	-	-
215			M	Chinese	17	24	No	Yes	Yes	Yes	Seborrhea, acne, watery diarrhea	periosteal overgrowth of the long bones	Compound heterozygous	c.664G>A c.1634delA	No	-	-
216			M	Chinese	19	42	No	Yes	Yes	Yes	Seborrhea, acne, watery diarrhea	periosteal overgrowth of the long bones	Heterozygous	c.861+2T>C	No	-	-
217			M	Chinese	17	17	Yes	Yes	Yes	Yes	Seborrhea Acne	periosteal overgrowth of the long bones	Heterozygous	c.1065dupA	No	-	-
218	Zhang Z [76]	2013	M	Chinese	18	27	Yes	Yes	Yes	Yes	Recurrent blepharitis	periosteal overgrowth of the long bones	Heterozygous Heterozygous missense	c.235-1G>T c.656C>T	No	-	-
219	Zhang Z [77]	2012	M	Chinese	-	24	-	Yes	Yes	Yes	-	Periostosis	Homozygous	c.97-1G>A	Yes	-	-
220			M	Chinese	-	27	-	Yes	Yes	Yes	Stomach hemorrhage due to a gastric ulcer	Periostosis	-	c.764G>A c.1634delA	No	-	-
221			M	Chinese	-	21	-	Yes	Yes	Yes	-	Periostosis	-	c.664G>A c.940+1G>A	No	-	-
222	Busch J [78]	2012	M	Japanese	20	53	-	Yes	-	Yes	Arthralgia	Periostosis	Heterozygous	c.940+1G>A c.1668G4C	-	-	-
223			M	Japanese	-	21	-	-	-	Yes	-	-	Homozygous	c.940+1G>A	Yes	-	-
224			M	Japanese	-	19	-	-	-	Yes	-	-	Homozygous	c.940+1G>A	Yes	-	-
225			M	Indian	25	27	-	Yes	Yes	Yes	Seborrhea	-	Homozygous	c.1292delC	Yes	-	-
226			M	Indian	17	26	Yes	Yes	-	Yes	Pain and swelling in ankle and knee	-	Homozygous	c.763G>A	Yes	-	-
227			M	Indian	17	28	Yes	Yes	-	Yes	Pain and swelling in ankle and knee	-	Homozygous	c.763G>A	Yes	-	-
228	Seifert W [79]	2012	M	Turkish	-	21	Yes	Yes	Yes	Yes	Arthralgia	Periostosis	Homozygous	c.830_831insT	-	-	-
229			M	Turkish	-	19	Yes	Yes	Yes	Yes	Arthralgia	Periostosis	Homozygous	c.830_831insT	-	-	-
230			M	Turkish	-	7	-	-	-	-	-	-	Homozygous	c.830_831insT	-	-	-
231			M	Turkish	-	40	-	-	-	Yes	-	-	Hetrozygous	c.830_831insT	-	-	-
232			M	Iraqi	-	38	Yes	Yes	Yes	Yes	Arthralgia	Periostosis	Homozygous	c.1670T>C c.1670T>C	-	-	-
233			M	Dutch	-	28	-	-	-	Yes	-	-	Hetrozygous	c.754C>T	-	-	-
234	Diggle CP [80]	2012	M	Hispanic (Colombia)	19	49	-	Yes	Yes	Yes	Anemia, myelofibrosis	Periostosis	-	c.1259G>T c.1259G>T	Yes	-	-
235			M	Chinese	16	-	-	Yes	Yes	Yes	-	Periostosis	-	c.941-1G>A c.1517C>A	-	-	-
236			M	Turkish	-	21	-	Yes	Yes	Yes	Anemia, myelofibrosis	Periostosis	-	c.542G>C c.542G>C	-	-	-
237			M	Dutch	-	-	-	Yes	Yes	Yes	-	Periostosis	-	c.1333C>T	-	-	-
238			M	French	-	-	-	-	-	-	-	-	-	c.290G>A c.940+2T>A	-	-	-
239			M	North African	-	16	-	-	-	-	Myelofibrosis	-	-	c.664G>A c.664G>A	-	-	-
240			M	North African	-	17	-	-	-	-	Myelofibrosis, hyperplastic gastropathy	-	-	c.253A>T c.253A>T	-	-	-
241			M	Dutch	-	19	-	-	-	-	-	-	-	c.1105+4A>G c.1105+4A>G	-	-	-
242			M	Kabardin (Caucasus)	-	24	-	-	-	-	-	-	-	c.838C>T c.1693T>G	-	-	-
243			M	Italian	17	-	-	-	-	-	-	-	-	c.310G>T c.310G>T	-	-	-
244			M	Algerian	14	-	-	-	-	-	-	-	-	c.724+1G>T c.724+1G>T	-	-	-
245			M	Turkish	-	-	-	-	-	-	Profound anemia, pancytopenia, myelofibrosis	-	-	c.542G>A c.542G>A	-	-	-
246			M	Italian	14	15	-	-	-	-	-	-	-	c.611C>T c.611C>T	-	-	-

Upon reviewing the collected data, we documented 246 patients diagnosed with PDP due to mutations in the SLCO2A1 gene [6,8,12-80]. Our patient’s c.563A>G variant has not been previously reported as a pathogenic mutation associated with PDP, making it a novel mutation. The most frequently observed variant was c.940+1G>A. The male-to-female ratio was approximately 32:1 (with 92.3% of patients being male), which is higher than the commonly reported 7:1 ratio in PDP cases.

The ethnic breakdown of the reported cases revealed the following distribution: Chinese (53.3%) (n=131), Japanese (7.3%) (n=18), Korean (4.9%) (n=12), Turkish (3.2%) (n=8), Indian (2.4%) (n=6), and Pakistani (2.4%) (n=6). This supports previous findings indicating that PDP is most commonly prevalent in Asian populations.

Interestingly, the age of diagnosis for SLCO2A1 mutations causing PDP was relatively consistent between two age groups: ≤25 years (37%) (n=91) and 25-45 years (30%) (n=74), with 7% (n=17) of patients diagnosed at ≥45 years of age. Regarding the age of symptom onset, the majority of patients (33%) (n=82) experienced symptoms before the age of 20. In a previously published review, the onset of symptoms typically occurs between the ages of 12 and 18 years [5]. However, hypothetically, this can vary based on factors such as the homozygosity and location of genetic mutations, as well as the underreporting of cases that may change this range. Due to the rarity of the disease and the overlap of its manifestations with those of other conditions, PDP may frequently be misdiagnosed as secondary hypertrophic osteoarthropathy, acromegaly, thyroid acropachy, or other rheumatologic diseases.

The clinical presentation of PDP among the patients in our review showed the following distribution: hyperhidrosis (41.1%) (n=101), pachyderma/thickening of the skin on the face and scalp (74.4%) (n=183), and digital clubbing (82.5%) (n=203). Interestingly, consanguinity was present in 29.7% (n=73) of patients, which is notably higher than the 8.5% (n=21) with consanguinity. The most common mutation type observed in the SLCO2A1 gene was homozygous (39%) (n=97), followed by compound heterozygous (15%) (n=38) and heterozygous (13%) (n=32).

In our review, several observations and limitations were noted. A large number of studies were excluded because they were published in Chinese and Japanese, with the most commonly reported ethnicities in our table being Chinese and Japanese. This means that the percentage of these ethnicities would likely be even higher if the excluded studies had been included. Additionally, many studies diagnosed PDP solely based on clinical presentation, excluding other potential causes without performing genetic testing, and these studies were excluded from our review. This shows that mutations in the SLCO2A1 gene may be underreported, indicating the need for further research. A significant number of patients in our search were found to have mutations in the HPGD gene, which is likely more prevalent than SLCO2A1 mutations and has been more extensively researched. Lastly, many patients diagnosed with CEAS mutation did not exhibit the clinical features of PDP, underscoring the need for further research and better documentation to enhance our understanding of the disorder and its associations.

The management of PDP is primarily symptomatic, focusing on improving the patient's quality of life. Treatment options include non-steroidal anti-inflammatory drugs (NSAIDs) and corticosteroids [81]. In a systematic review published in 2017, 70% of patients treated with NSAIDs showed improvement in arthritis and arthalgia symptoms [82]. A study published in 2018 explored the use of hydroxychloroquine in a patient with PDP, resulting in a decrease in pain and improvement in the patient’s skin condition [16]. Additionally, bisphosphonates have been studied in both primary and secondary hypertrophic osteoarthropathy, with a recent meta-analysis suggesting their safety and efficacy. A total of 88.3% of patients with hypertrophic osteoarthropathy (primary and secondary) showed improvement in pain or arthritis [83]. Recently, a somatostatin analogue, lanreotide autogel (60 mg intramuscularly, administered once monthly for 12 months), was used for the first time in a patient with PDP. Notable improvements were observed, particularly with regard to excessive sweating and arthralgia [81]. Further research is warranted to evaluate the efficacy of somatostatin analogues in PHO. Biologic agents such as infliximab have also been trialed in refractory cases of PDP, though outcomes have been variable [84]. In severe, refractory cases of PDP that do not respond to medical therapy, surgical interventions may be considered [81].

## Conclusions

PDP is a rare genetic disorder that is prevalent in the Asian population. Patients commonly present with digital clubbing, pachydermia, and periostitis. Genetic testing of the HPGD and/or SLCO2A1 genes is used for a definitive diagnosis. While the primary treatment involves the use of NSAIDs, other therapeutic options have been explored, and further research is necessary to evaluate their effectiveness.
